# Retinal vascular parameters as AI-driven biomarkers for pulse wave velocity assessment: a telemedicine strategy for cardiovascular risk assessment

**DOI:** 10.1080/07853890.2026.2682652

**Published:** 2026-06-26

**Authors:** Yuelin Wang, Ying Che, Jinghao Qu, Yi Qu, Peng Wang, Jing Hong

**Affiliations:** a Department of Ophthalmology, Peking University Third Hospital, Beijing, China; b Medical Examination Center, Peking University Third Hospital, Beijing, China

**Keywords:** Retinal biomarkers, pulse wave velocity, artificial intelligence, cardiovascular risk

## Abstract

**Background:**

Pulse wave velocity (PWV), known as the gold standard for evaluating arterial stiffness, is limited by device dependence. This study aims to explore the feasibility of retinal parameters to assess PWV based on artificial intelligence (AI), providing a new approach for remote and convenient cardiovascular risk assessment.

**Methods:**

This retrospective study leveraged both cross-sectional and longitudinal data, enrolling patients from Peking University Third Hospital who underwent fundus photography and PWV testing simultaneously over five consecutive years. An optimised AI system automatically quantified retinal vascular parameters, optic morphological features, and lesion counts. Correlation analyses and multivariate linear regression models were employed using the training set to assess associations between retinal features and brachial-ankle PWV (baPWV) or ankle-brachial index (ABI), with validation performed in the test set.

**Results:**

In the training set of 3,088 visits, baPWV and ABI showed significant correlations with retinal vascular morphology (including mean vessel density, arteriolar-to-venular ratio, regional curvature, fractal dimension), optic disk/cup parameters (disk area, circularity), and lesion counts (exudative and drusen) (all *p* < 0.001). Elevated baPWV was independently associated with older age, higher blood pressure, lower arteriolar-to-venular ratio, larger disk area, and increased soft exudates and drusen (*F* = 503.864, *p* < 0.001). Higher ABI was linked to older age, higher diastolic pressure, lower arteriolar-to-venular ratio, decreased vessel density, and fewer hard exudates (*F* = 71.368, *p* < 0.001). Validation demonstrated good agreement between predicted and actual baPWV (ICC = 0.762, *p* < 0.001), while agreement for ABI was moderate (ICC = 0.410, *p* < 0.001). Longitudinal analysis further revealed that baPWV changes correlated with fractal dimension, vessel density, arteriolar/venular ratio, and disk circularity, whereas ABI changes were associated with fractal dimension, vessel diameter, vessel density, arteriolar diameter, and drusen count (all *p* < 0.001).

**Conclusion:**

This large-scale study demonstrates significant associations between AI-derived retinal features and baPWV/ABI, identifying robust predictive markers. The proposed AI-based retinal analysis offers a non-invasive, scalable, and remote screening paradigm for arterial stiffness and cardiovascular risk assessment.

## Key messages

- AI-derived retinal parameters, including vascular morphology and optic disk features, show significant correlations with arterial stiffness and vascular health, offering a non-invasive alternative to traditional PWV assessment.
- Key retinal predictors of elevated baPWV include increased blood pressure, age, lesion counts, reduced arteriolar-to-venular ratio, enabling AI-based cardiovascular risk evaluation.
- Longitudinal analysis confirms that changes in retinal vascular metrics (e.g. fractal dimension, vessel density) track with baPWV/ABI progression, supporting AI-powered retinal screening as a scalable tool for remote arterial stiffness monitoring.

## Introduction

Cardiovascular disease (CVD) has become a major global public health challenge [1]. Notably, nearly 80% of cardiovascular events can be effectively prevented through early lifestyle interventions [2]. The necessity of early CVD screening plays a crucial role in reducing disease-related mortality and alleviating the global disease burden.

Pulse wave velocity (PWV) is a noninvasive and accurate method for measuring arterial stiffness [3]. It assesses cardiovascular health by measuring the speed of the pulse wave generated during cardiac contraction through the arterial system. The stiffer the blood vessels, the faster the pulse wave propagates [4]. Studies have shown that PWV can effectively predict cardiovascular mortality and all-cause mortality [5,6] and is considered the gold standard method for evaluating arterial stiffness. Some clinical trials have shown that abnormal PWV can predict cardiovascular events 5–8 years earlier than traditional risk factors [6,7]. Besides, PWV also shows a significant positive correlation with high‑sensitivity cardiac troponin T (hs‑cTnT) concentration, a biochemical marker of myocardial injury, even in the absence of an acute clinical context, particularly in hypertensive patients [8]. Therefore, early detection of PWV abnormalities allows for targeted interventions to delay the progression of CVDs.

However, current PWV measurements rely on high-precision specialised equipment and strict standardised operating procedures, posing significant challenges to its application in remote areas and regions with limited medical resources [9]. On the one hand, advanced detection equipment is costly and requires regular maintenance, making it difficult to afford. On the other hand, the measurement process is technically demanding—operators must precisely position blood vessels, synchronise multi-site signals, and filter physiological interference, all of which require specialised training [10]. These limitations hinder the widespread adoption of PWV testing in primary healthcare settings, highlighting the urgent need to develop portable, low-cost, and easy-to-use alternative technologies.

The retinal vasculature is the only microcirculatory system in the human body that can be directly observed [11]. The morphological changes of retinal vessels can reflect the pathological processes of various systemic vascular diseases [12]. In recent years, artificial intelligence-based retinal image analysis technology has advanced rapidly [13]. This technology not only improves the efficiency of early screening but also enables dynamic monitoring of disease progression, providing evidence for clinical decision-making.

Current research evidence on the association between retinal vasculature and PWV remains insufficient, primarily reflected in the following two aspects [14–17]: (1) Lack of quantitative vascular measurement analysis. Existing studies have found some associations between PWV with fundus diseases of age-related macular degeneration (AMD), glaucoma, and retinal vein occlusion (RVO), but lack quantitative measurements and explainability of retinal vessels. (2) Lack of longitudinal research data support. Most existing studies are cross-sectional in design. Due to the influence of confounding factors arising from inter-individual differences, there is currently a lack of research that establishes more sensitive methods for assessing continuous changes in PWV through repeated measurements of the same subjects.

**Figure 1.**
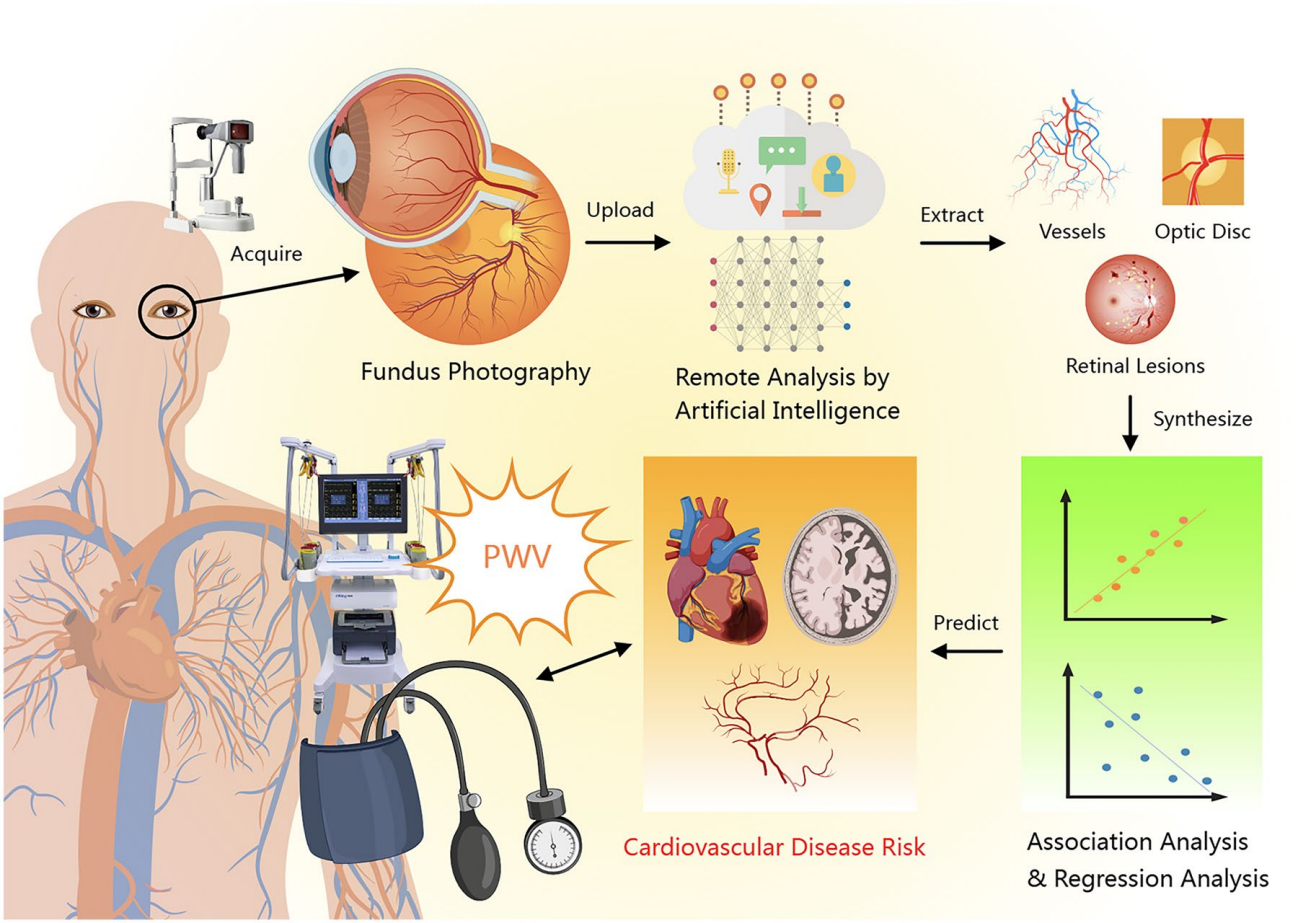
Fundus photography-based remote PWV assessment for cardiovascular risk prediction. This innovative approach captures retinal images using any fundus camera (including portable smartphone-based devices), then leverages cloud-based AI to analyse retinal vasculature, optic disk features, and lesions, combined with statistical modelling for fast and convenient cardiovascular risk assessment (This diagram is created with MedPeer (medpeer.cn)).

This study, both cross-sectional and longitudinal data, aims to explore the association between PWV and retinal vascular morphology and its predictive value. The study intends to use artificial intelligence technology to quantify retinal parameters, including retinal vascular morphological features, optic disk structural parameters, and retinal lesions, and analyse their correlation with PWV. Furthermore, by using multi-timepoint follow-up data from the same participant, the study will construct correlations between retinal vascular features and PWV, and establishing a noninvasive PWV evaluation system based on fundus imaging. Figure 1 illustrates the novel paradigm of fundus photography-based remote PWV assessment for cardiovascular risk prediction proposed in this study.

## Methods

### Study design and population

**Figure 2.**
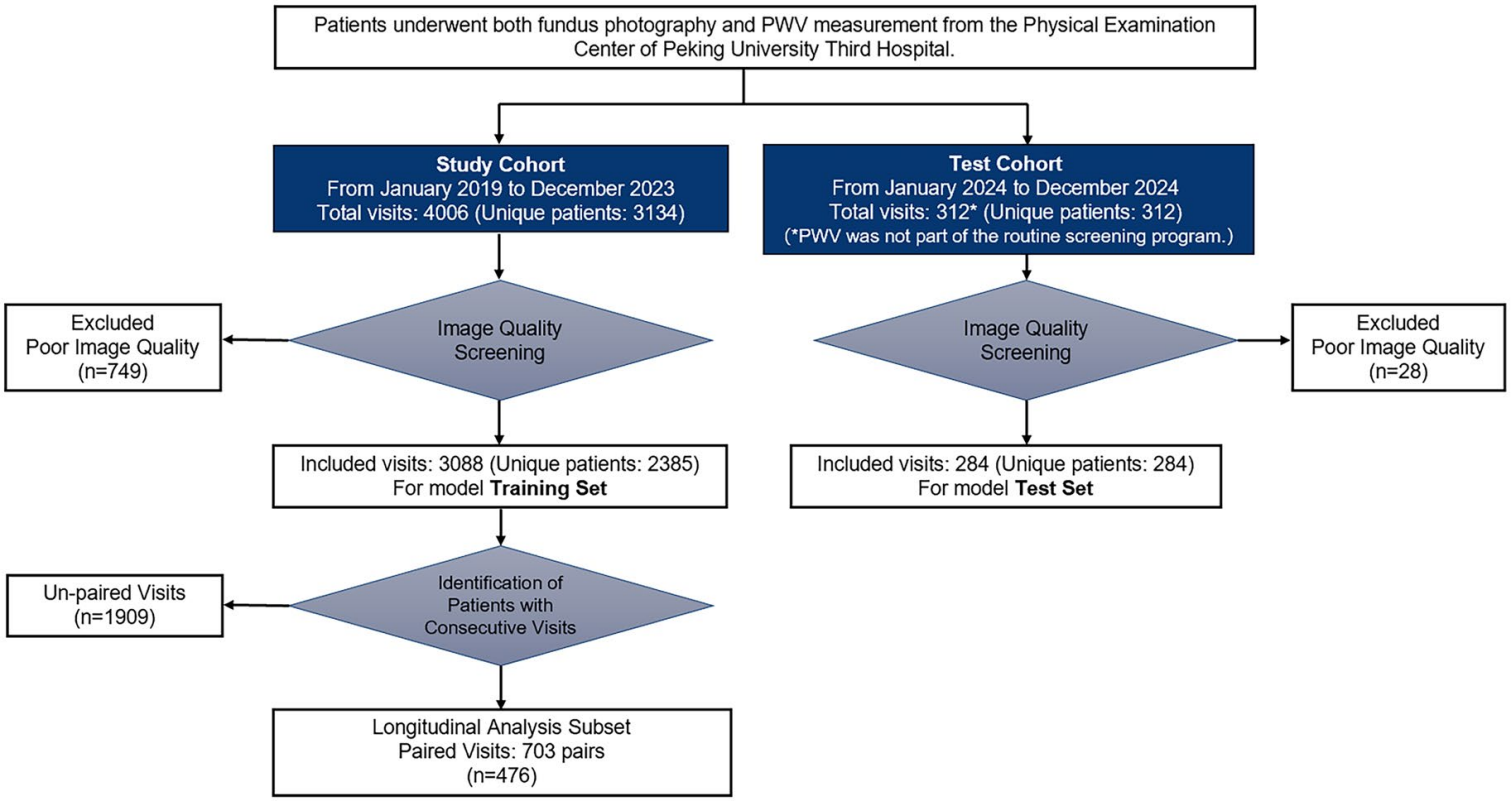
Flowchart of included patients.

This study is a retrospective longitudinal cohort analysis of patients who underwent both fundus photography and PWV measurement at the Physical Examination Centre of Peking University Third Hospital. The training set was collected from January 2019 to December 2023, while an independent test set was derived from visits between January and December 2024. The included flowchart is shown in Figure 2.

The study protocol was approved by the Institutional Ethics Committee of Peking University Third Hospital, and informed consent was waived due to the retrospective nature of the analysis (Ethics Review Number: (2025)134-01) by the Institutional Ethics Committee of Peking University Third Hospital. All procedures followed the principles of the Declaration of Helsinki.

The inclusion criteria of this study were: (1) age ≥ 18 years at health examination; (2) completion of both non-mydriatic fundus photography and PWV measurement within the same outpatient visit and within a 12 h window; and (3) availability of complete demographic information (age, sex) and blood pressure measurement. Exclusion criteria were: (1) poor quality fundus images (significant motion artefacts, inadequate illumination/focus, or media opacities) precluding reliable retinal vascular assessment; (2) history of major ocular diseases or surgeries (e.g. diabetic retinopathy, retinal vein/artery occlusion, glaucoma, age related macular degeneration); (3) known cardiac arrhythmias, particularly atrial fibrillation; (4) pregnancy; (5) acute febrile illness or any acute cardiovascular event within 3 months prior to examination; and (6) history of major limb amputation or peripheral revascularization that precludes reliable brachial ankle PWV measurement.

### Examinations

Demographic and clinical characteristics (including age and sex) were extracted from electronic medical records.

### Fundus photography

Patients were seated in a dimly lit room, and non-mydriatic 45-degree fundus photographs were taken by experienced technicians using a Canon CR-DGi camera (Canon, Japan). Each image ensured coverage of the macular and optic disk regions.

### PWV measurement

PWV and ankle-brachial index (ABI) were assessed with the Omron BP-203RPEIII arteriosclerosis detection device (Omron Healthcare, Kyoto, Japan), a dedicated blood pressure pulse wave monitor. Before measurement, participants rested for 5 min in a room maintained at 22–25 °C. Measurements were taken in the supine position, with cuffs placed on the limbs (2–3 cm above the elbow for the arm and 1–2 cm above the medial ankle for the leg) and connected to ECG and phonocardiogram monitoring devices. Each participant underwent three consecutive bilateral PWV measurements, and the mean value was calculated [18].

Brachial-ankle PWV (baPWV) was derived using the formula:

baPWV (m/s)=L/T

(*L* is the body surface distance between the brachial and ankle arteries, and *T* is the pulse wave transit time.)baPWV values exceeding 14 m/s indicate early vascular ageing and elevated risks of hypertension, stroke, and cardiac events [19].

The ABI was calculated using the formula:

$$\text{ABI}=\text{Systolic pressure at brachial artery }\left(\text{mmHg}\right)/\text{Highest systolic pressure at ankle }\left(\text{mmHg}\right)$$

(ABI as the ratio of systolic blood pressure in the ankle (tibial or dorsalis pedis artery) to that in the brachial artery)

ABI quantifies blood flow efficiency to the lower extremities. Values below 0.90 indicate significant arterial blockage, while abnormally high values (>1.40) suggest arterial calcification [19,20].

### AI-based retinal morphology analysis

The *EVISION model*, an AI-driven framework based on human visual bionic mechanisms and deep learning—was employed for retinal vessel segmentation. This model utilises intelligent image processing algorithms to accurately identify and quantify lesion features. It has been validated across multiple studies and demonstrates high accuracy in image measurement and recognition; performance evaluation yielded results of 0.9652 accuracy, 0.7247 sensitivity, and 0.9605 specificity [21–23]. The following parameters were extracted in this study:

1. Vessel Diameters: Mean arterial diameter (overall and within 0.5–1.0 PD, 1.0–1.5 PD, and 1.5–2.0 PD regions), mean venous diameter (overall and within 0.5–1.0 PD, 1.0–1.5 PD, and 1.5–2.0 PD regions), arteriole-to-venule diameter ratio (AVR), fractal dimension, vessel density.
2. Optic Disk Information: Disk area, circularity, tilt angle, and distance to the macula.
3. Retinal Lesions: Total number of retinal haemorrhages, hard exudates, and soft exudates.

### Longitudinal data setup

For longitudinal analysis, each patient’s consecutive measurements were paired. The correlation between changes in baPWV/ABI and changes in retinal parameters was assessed to determine whether baPWV/ABI progression was associated with retinal morphological alterations over time.

### Data analysis

All analyses were performed using SPSS 26.0, with a significance level of α = 0.05. Normality was assessed using the Shapiro-Wilk test. Continuous variables were reported as mean ± standard deviation (SD) or median (interquartile range, IQR) as appropriate, while categorical variables were presented as counts (percentages). Pearson or Spearman correlation tests were used to evaluate the relationship between baPWV/ABI and retinal features, depending on data distribution. Longitudinal changes in matched pairs were analysed for correlations between PWV/ABI and retinal parameter variations. Multiple linear regression was performed to assess associations between PWV, ABI, and retinal morphological parameters, with results reported as beta coefficients with 95% confidence intervals (CI). Since age and blood pressure are currently recognised as key indicators associated with PWV, metrics for age, systolic blood pressure, and diastolic blood pressure were added to the multiple linear regression model. The obtained model was subsequently tested in the test set. The predicted values generated by the model from the training dataset were compared with the actual measured baPWV and ABI values. This comparison was performed using the Intraclass Correlation Coefficient (ICC). Additionally, Bland-Altman plots were constructed to assess and visualise the diagnostic agreement.

## Results

### Baseline information

This study ultimately included 4,006 visits (3,134 individuals) from participants examined at the Health Examination Centre of Peking University Third Hospital between January 2019 and December 2023 in the Study Data. After manually screening out images of poor quality, 3,088 visits (2,385 individuals) were included in the analysis. Among them, consecutive measurements from the same patient were treated as matched pairs, resulting in 703 matched pairs (476 individuals) for paired analysis. In the test set, a total of 312 visits (corresponding to 312 patients) were initially included. Following a manual assessment of image quality, 284 patients were ultimately retained for final analysis as the test data. (see Table 1, Figure 2)

**Table 1.** Demographic table of included patients.

	Training set (*N* = 3088)	Test set (*N* = 284)
Male (%)	1819 (58.91)	186 (65.50)
Age, years old	48.53 ± 10.72	48.98 ± 10.05
Average baPWV, m/s	1.352 ± 0.257	1.344 ± 0.230
Average ABI	1.29 ± 9.10	1.15 ± 0.07
Systolic pressure, mmHg	125.46 ± 16.56	122.79 ± 15.40
Diastolic pressure, mmHg	77.70 ± 11.15	75.54 ± 10.52

Abbreviations: ABI: ankle-brachial index; baPWV: brachial-ankle pulse wave velocity.

**Figure 3.**
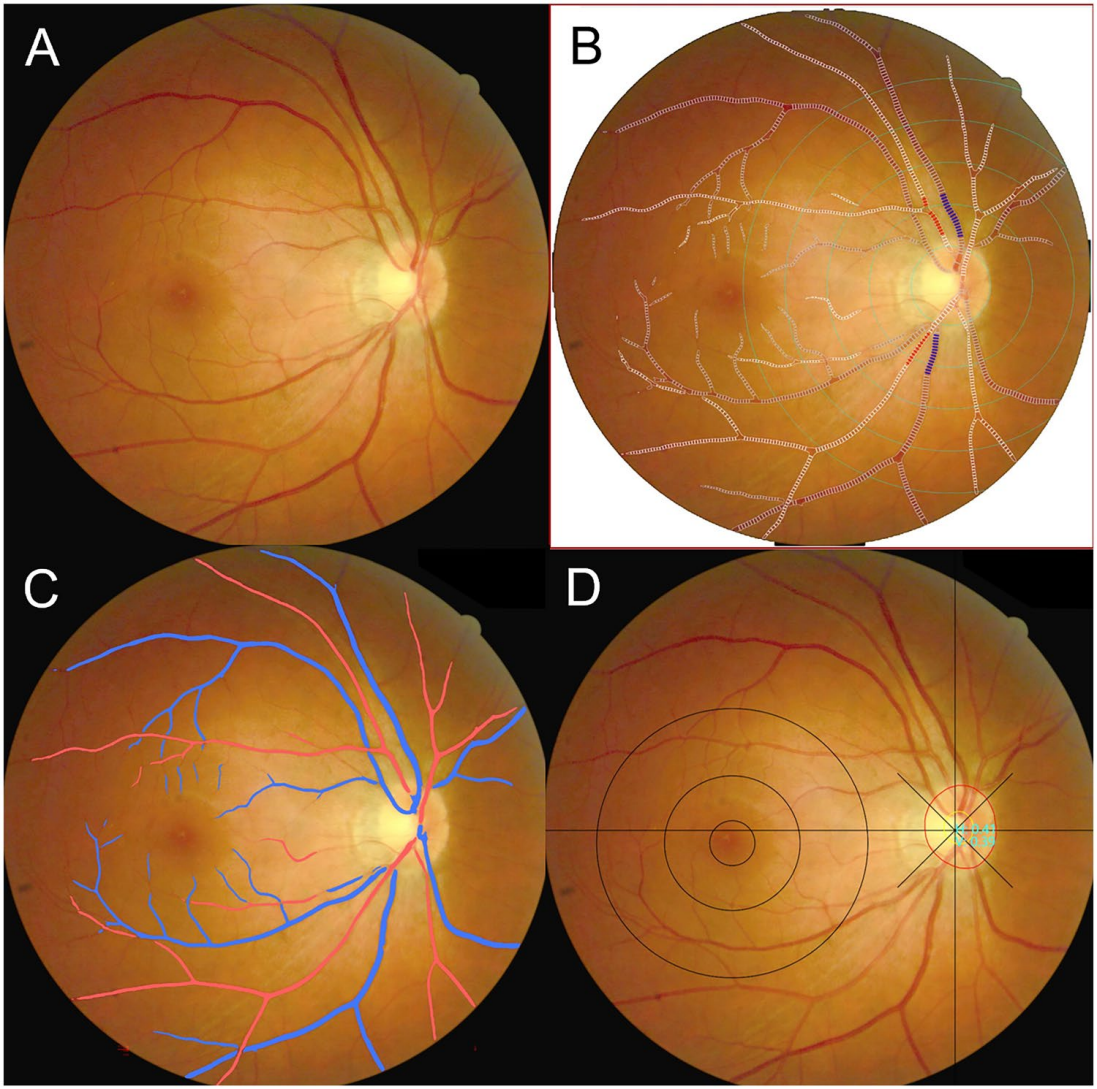
AI-based extraction of retinal vascular information. (A) shows the patient’s normal fundus image; (B) demonstrates the division of different vascular regions at 0.5PD (optic disk diameter) intervals from the optic disk using AI; (C) presents the automatic extraction of arteries and veins from the patient’s retinal vascular system by AI; (D) displays the results of automatic recognition and labelling of the optic disk and macular region by AI.

Of the 2,385 patients included in the Study Data, the mean age was 48.31 ± 11.04 years, with 1,332 males and 1,053 females. Among them, 545 had hypertension, and 165 had diabetes. For the 3,088 visits analysed, the mean bilateral baPWV was 1.352 ± 0.257 m/s, and the mean ABI was 1.29 ± 0.10. A comprehensive analysis of retinal vascular features, optic disk morphology, and systemic vascular indicators was conducted for the included patients. The AI-based extraction of retinal vascular information is shown in Figure 3.

### Retinal vascular morphology analysis of included patients

The results showed that the mean fractal dimension of retinal vessels was 1.49 ± 0.07, with regional variations in vessel diameter—the largest diameter was observed in the 0.5–1.0 PD region (87.50 ± 8.87 μm). The mean arterial diameter (63.16 ± 7.76 μm) was smaller than the venous diameter (68.24 ± 12.92 μm). The mean vessel tortuosity was 0.13 ± 0.30, with veins (0.15 ± 0.33) exhibiting higher tortuosity than arteries (0.11 ± 0.25). Regarding optic disk morphology, the mean area was 2.46 ± 0.56 mm^2^, and the cup-to-disk ratio (by area) was 0.21 ± 0.07. Among retinal lesions, haemorrhages (0.05 ± 0.41) and microaneurysms (0.11 ± 0.80) were predominantly distributed in the temporal region, while exudates were relatively rare. These results provide essential baseline data for studying the association between retinal and systemic vascular health, demonstrating the feasibility of this retinal analysis system. Overall, the retinal vascular characteristics of the included population were within normal ranges.

### Correlation between PWV and vascular morphology

**Figure 4.**
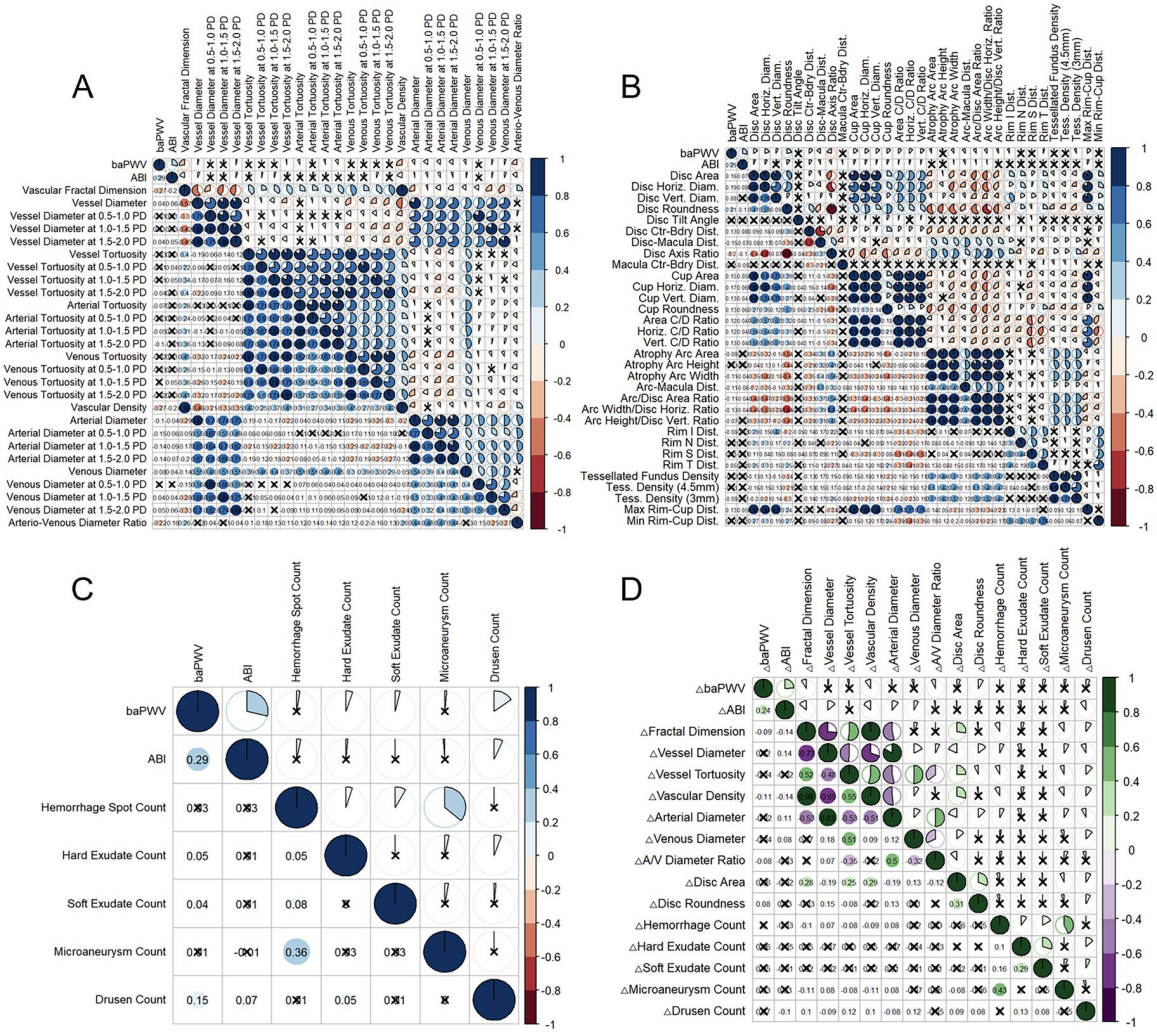
Correlation analysis between PWV and retinal vasculature, optic disk structure, and retinal lesions, along with longitudinal correlation analysis. (A) PWV vs retinal vasculature; (B) PWV vs optic disk structure; (C) PWV vs retinal lesions; (D) Longitudinal comparison showing correlations between changes in baPWV/ABI and variations in retinal vasculature, optic disk characteristics, and retinal lesions over time. The row and column headers indicate the variables being compared. In the upper-right quadrant, the size of each pie chart represents the correlation coefficient (‘r’), with an “X” denoting a non-significant association. In the lower-left quadrant, the colour of each circle indicates the correlation coefficient (‘r’), with an “X” again marking non-significant associations. The colour bar on the far right represents the magnitude of the correlation coefficient. Abbreviations: ABI: ankle-brachial index; Arc: Atrophy Arc; baPWV: brachial-ankle pulse wave velocity; C/D: Cup-to-Disk Ratio; Cup: Optic Cup; Ctr-Bdry: Centre-to-Boundary; Diam.: Diameter; Disk: Optic Disk; Dist.: Distance; Horiz.: Horizontal; Tess.: Tessellated; Vert.: Vertical.

Analysis of the correlation between vascular morphological parameters and vascular function indicators (baPWV and ABI) revealed that the fractal dimension of blood vessels was significantly negatively correlated with both baPWV (*r* = −0.265) and ABI (*r* = −0.196) (*p* < 0.01) (see Figure 4A). Vessel diameter analysis showed a weak positive correlation between overall mean vessel diameter and baPWV (*r* = 0.044, *p* = 0.015), while arterial diameter exhibited a significant negative correlation with baPWV (*r* = −0.100, *p* < 0.01), particularly in the 1.5–2.0 PD region (*r* = −0.144, *p* < 0.01). Vessel tortuosity in the 1.5–2.0 PD region showed a significant negative correlation with baPWV (*r* = −0.036, *p* = 0.043), with arterial tortuosity displaying a more pronounced negative correlation (*r* = −0.068, *p* < 0.01). Additionally, vascular density was significantly negatively correlated with both baPWV (*r* = −0.271) and ABI (*r* = −0.198) (*p* < 0.01), and the arteriole-to-venule diameter ratio also showed negative correlations with baPWV (*r* = −0.223) and ABI (*r* = −0.156) (*p* < 0.01). Compared to baPWV, ABI exhibited weaker associations with vascular parameters, though the trends were similar, possibly reflecting ABI’s greater focus on assessing lower limb arterial occlusion, whereas baPWV is more sensitive to systemic arterial stiffness.

### Correlation between PWV and optic disc morphological features

Analysis of the correlation between optic disk morphological features and vascular function indicators (baPWV and ABI) revealed significant associations between optic disk structural parameters and arterial stiffness (see Figure 4B). The results showed that optic disk area (*r* = 0.159, *p* < 0.01), horizontal diameter (*r* = 0.192, *p* < 0.01), and circularity (*r* = 0.214, *p* < 0.01) were significantly positively correlated with baPWV, while the optic disk long-to-short axis ratio (*r* = −0.205, *p* < 0.01) exhibited a significant negative correlation. Notably, optic cup-related parameters (including area, horizontal diameter, and vertical diameter) were all significantly positively correlated with baPWV (*p* < 0.01), and cup-to-disk ratio metrics (area, horizontal, and vertical) showed similar trends (*p* < 0.01). Parameters related to peripapillary atrophy were generally negatively correlated with baPWV, with atrophy area (*r* = −0.078, *p* < 0.01) and width (*r* = −0.110, *p* < 0.01) showing the most significant correlations. In ABI analysis, although the correlation strengths were weaker than those for baPWV, significant positive correlations were observed for optic disk area (*r* = 0.067, *p* < 0.01), horizontal diameter (*r* = 0.082, *p* < 0.01), and circularity (*r* = 0.094, *p* < 0.01). The density of tessellated fundus within a 4.5 mm diameter region centred on the optic disk showed significant correlations with both baPWV (*r* = −0.077, *p* < 0.01) and ABI (*r* = 0.052, *p* = 0.004), suggesting that retinal microvascular changes may be linked to systemic vascular function.

### Correlation between PWV and retinal lesions

Analysis of the correlation between retinal lesion characteristics and vascular function indicators (baPWV and ABI) revealed differential associations between different types of retinal lesions and arterial stiffness (see Figure 4C). In baPWV analysis, soft exudates (*r* = 0.068, *p* < 0.01) and drusen (*r* = 0.150, *p* < 0.01) exhibited the strongest positive correlations, while hard exudates also showed a significant positive correlation (*r* = 0.053, *p* = 0.003). In ABI analysis, significant positive associations were observed for soft exudates (*r* = 0.035, *p* = 0.049) and drusen (*r* = 0.065, *p* < 0.01).

### Retinal predictors of PWV

Multivariate linear regression models were conducted to evaluate retinal predictors associated with baPWV and ABI (see Table 2). Given the potential confounding effect of age and blood pressure, it was included as a covariate in the analysis. Multivariate regression identified key predictors: elevated baPWV was independently associated with older age, higher blood pressure, lower arteriolar-to-venular ratio, larger disk area, and increased soft exudates and drusen (*F* = 503.864, *p* < 0.001). Higher ABI was linked to older age, higher diastolic blood pressure, lower arteriolar-to-venular ratio, decreased vessel density, fewer number of hard exudates (*F* = 71.368, *p* < 0.001).

**Table 2.** Multivariate analysis of retinal image characteristics for baPWV and ABI.

	Unstandardized coefficients		Standardized coefficients	*t*	*p*	*F*	D-W
	B	95% CI	Beta				
**baPWV**						503.864, (*p* < 0.001)	1.983
(Constant)	−3.769	−94.643 to 87.105	–	−0.082	0.934		
Systolic blood pressure	6.124	5.505–6.743	0.395	19.386	0.000		
Age	9.751	9.113–10.389	0.407	29.963	0.000		
Diastolic blood pressure	2.427	1.540–3.314	0.105	5.362	0.000		
Average total number of soft exudates	428.900	221.054–636.746	0.050	4.042	0.000		
Average arteriovenous diameter ratio	−156.474	−235.930 to −77.018	−0.049	−3.860	0.000		
Average optic disc area (unit: mm²)	16.381	5.109–27.653	0.036	2.848	0.004		
Average total number of drusen	11.434	0.630–22.238	0.026	2.075	0.038		
**ABI**							
(Constant)	0.631	0.478–0.784	–	8.147	0.000	71.368, (*p* < 0.001)	1.978
Age	0.002	0.002–0.002	0.292	16.003	0.000		
Diastolic blood pressure	0.001	0.001–0.001	0.091	5.197	0.000		
Average arteriovenous diameter ratio	−0.079	−0.11 to −0.04	−0.085	−4.523	0.000		
Average total number of hard exudates	−0.011	−0.02 to −0.001	−0.038	−2.281	0.023		
Average vascular density	−1.332	−1.80 to −0.86	−0.324	−5.551	0.000		

Abbreviations: ABI: ankle-brachial index; baPWV: brachial-ankle pulse wave velocity.

The model was evaluated on the independent test set. Results demonstrated good agreement between model-predicted and measured baPWV, with an intraclass correlation coefficient (ICC) of 0.762 (95% CI: 0.708–0.806, *p* < 0.001). The agreement for ABI was moderate, with an ICC of 0.410 (95% CI: 0.309–0.502, *p* < 0.001). Bland-Altman analysis demonstrated good overall agreement between model-predicted and measured values for both baPWV and ABI (see Supplementary Figure 1). These findings support the feasibility of using AI-extracted fundus image markers to estimate baPWV and ABI, while also providing preliminary validation of the model’s generalisability in an external dataset.

### Longitudinal data analysis

The study results indicate that longitudinal changes in baPWV were significantly correlated with the average vascular fractal dimension, average vascular density, average arteriovenous diameter ratio, and average optic disk roundness. The longitudinal changes in ABI were significantly associated with the average vascular fractal dimension, average vascular diameter, average vascular density, average arterial mean diameter, and average total number of drusen (see Figure 4D).

## Discussion

This study found that features extracted from noninvasive, easily accessible retinal images using AI algorithms can effectively reflect the degree of arterial stiffness traditionally measured by specialised PWV devices.

In this study, retinal vascular features showed significant correlations with PWV and were important predictors of baPWV and ABI. Elevated baPWV was associated with older age, higher blood pressure, lower arteriolar-to-venular ratio, larger disk area, and more soft exudates/drusen. These associations strongly suggest that the pathological changes in the retinal microcirculatory network (e.g. rarefaction, arteriovenous disproportion) may share deep pathophysiological mechanisms with the stiffening process of central large arteries (e.g. the aorta). The study indicates that a baPWV >1.4 m/s signifies severely increased arterial stiffness and significantly elevated CVD risk (e.g. myocardial infarction, stroke), which aligns with our findings of retinal arterial narrowing and reduced AVR. The decreased retinal vascular density and altered arteriovenous ratio may reflect systemic endothelial dysfunction, microvascular rarefaction, and vascular remodelling—core processes in atherogenesis [24]. Notably, this study also found that higher ABI correlated with older age, higher diastolic blood pressure, lower arteriolar-to-venular ratio, reduced vessel density, and fewer hard exudates. Generally, both abnormally low and high ABI indicate large-vessel stenosis or stiffness: ABI <0.9 suggests lower limb ischaemia and arterial stiffness, while ABI >1.4 indicates vascular calcification [25,26]. In this study, reduced retinal vascular density and arterial diameter were associated with elevated ABI, possibly reflecting retinal vascular manifestations of calcification [27]. These findings suggest that retinal vascular morphological parameters could serve as potential biomarkers for assessing arterial stiffness and peripheral arterial disease (PAD), warranting further validation of their predictive value for cardiovascular events using longitudinal data.

Optic disk structural parameters were also associated with PWV. The study found that baPWV correlated with enlarged disk area, increased cup-to-disk ratio, expanded parapapillary atrophy, and geometric abnormalities, suggesting that systemic vascular stiffness may mechanically induce structural remodelling of the optic nerve head [28,29]. In contrast, ABI was primarily linked to reduced disk circularity, altered atrophy arc proportions, and increased macular distance, reflecting microcirculatory adaptations to peripheral ischaemia [30]. These findings indicate that optic disk morphology and border characteristics may relate to intraocular hemodynamic changes or vascular wall properties. Retinal imaging thus provides a unique and intuitive “visual window” for the early, noninvasive monitoring of systemic arterial stiffness.

Additionally, retinal lesions were associated with PWV and ABI. This study found ABI abnormalities (typically indicating lower limb arterial obstruction) significantly correlated with retinal microvascular pathologies, including increased soft exudates and drusen counts. Soft exudates suggest localised retinal ischaemia and lipid leakage due to blood-retinal barrier disruption, while drusen are linked to metabolic dysfunction and ageing in the choroid-Bruch’s membrane-RPE complex. These findings indicate that ABI-defined lower limb arterial obstructive disease shares specific pathological links with retinal ischaemic damage, vascular leakage, and degenerative changes. This provides a theoretical basis for using retinal imaging as a remote, convenient screening tool to identify high-risk PAD individuals.

This study innovatively employed longitudinal data analysis to further validate the retina as a dynamic monitoring window for PWV. Changes in baPWV and ABI over time were closely associated with retinal microvascular structural parameters: baPWV progression correlated significantly with mean vascular fractal dimension, vascular density, AVR, and optic disk circularity, while ABI changes were linked to mean fractal dimension, vessel diameter, vascular density, arterial diameter, and total drusen count. This finding not only expands the dimensions of traditional CVD risk assessment but also offers a clinically actionable screening tool—evaluating long-term PWV and ABI trends indirectly *via* fundus examination, particularly suitable for long-term follow-up in high-risk populations [31].

The key innovation of this study lies in demonstrating that AI-assisted fundus image analysis is significantly associated with arterial stiffness as measured by PWV. Our regression analysis further shows that AI-derived retinal vascular features can serve as a noninvasive, convenient tool for predicting PWV, thereby aiding in the assessment of arterial stiffness and contributing to the early prevention of cardiovascular disease risk. A major advantage is its ability to bypass the need for specialised PWV equipment, enabling large-scale screening in primary care settings, clinics, or even telemedicine settings. This establishes a “single retinal scan, multiple risk assessments” model, significantly expanding the reach and accessibility of preventive cardiovascular screening.

This study has some limitations. First, due to sample size limitations, the representativeness and statistical validity of research results may be affected. Second, current research conclusions are mainly based on observational data. Although a correlation between retinal parameters and changes in baPWV/ABI has been found, further validation of its causal relationship and predictive value is still needed through large-scale prospective cohort studies. In the future, it is necessary to establish a standardised retinal imaging analysis process through multi-centre collaborative research, and combine it with long-term follow-up data to provide a more reliable evidence-based basis for developing precise cardiovascular risk stratification strategies.

## Conclusion

This study validates and quantifies the strong link between AI-derived retinal features and cardiovascular risk markers (baPWV and ABI). Regression analysis identified key retinal predictors, while longitudinal data tracked dynamic changes in both vascular and retinal parameters, supporting retinal-based CVD risk prediction. This AI-powered approach uses routine, noninvasive fundus imaging, eliminating reliance on specialised PWV devices—enabling remote, convenient, and scalable screening.

## Supplementary Material

Supplementary Figure 1.docx

## Data Availability

The datasets supporting the conclusions of this article will be available from the corresponding author on reasonable request.
