# Supplementary material for: Retinal vascular parameters as AI-driven biomarkers for pulse wave velocity assessment: a telemedicine strategy for cardiovascular risk assessment
Source: Ann Med. 2026 Jun 26;58(1):2682652. doi: 10.1080/07853890.2026.2682652 (PMC13312835; doi:10.1080/07853890.2026.2682652)
Supplement: Supplementary Figure 1.docx [file IANN_A_2682652_SM3503.docx]

**
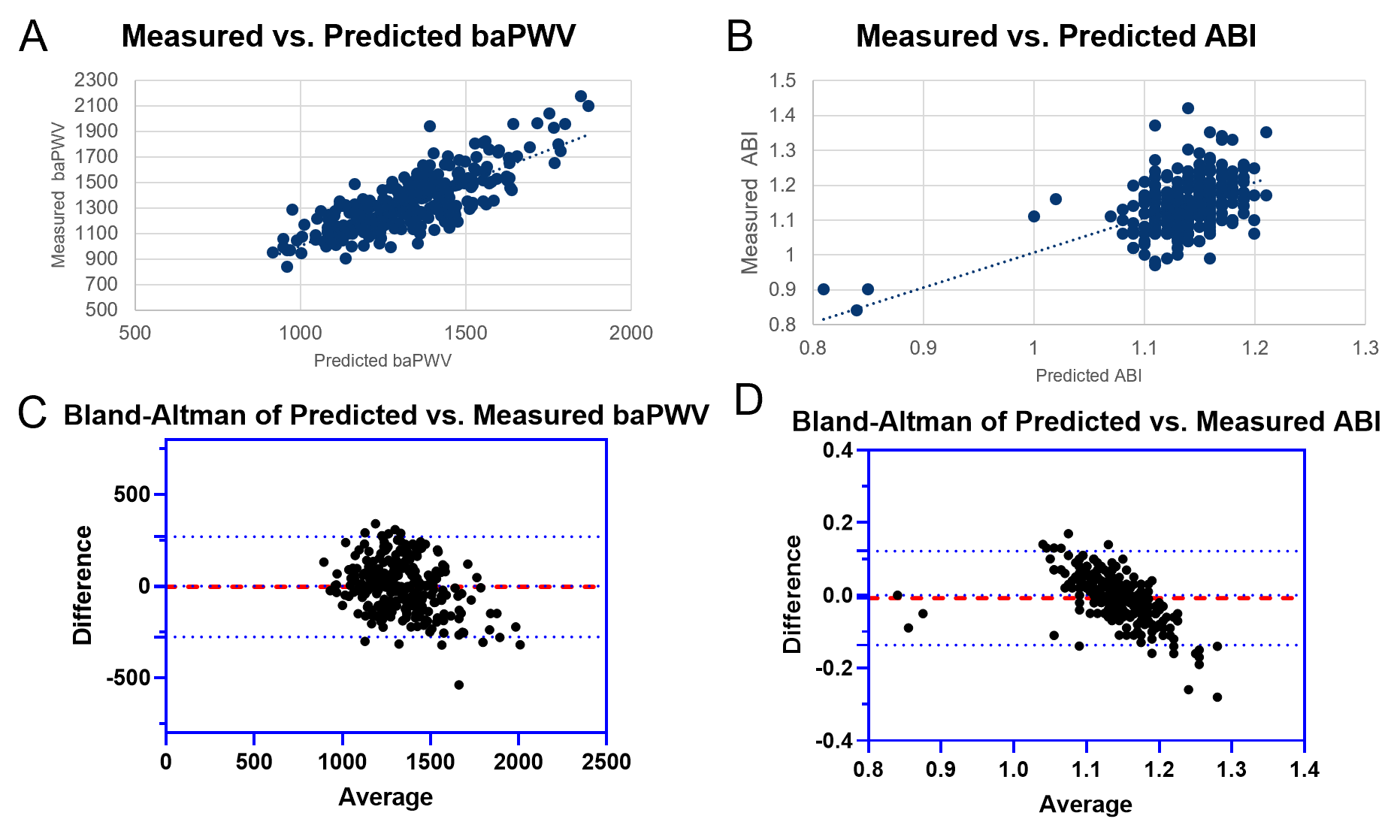
**

**Supplementary Figure 1. Predictive Agreement of baPWV and ABI in the Test Set.** A, Comparison between measured and predicted baPWV values; B, Comparison between measured and predicted ABI values; C, Bland–Altman plot of measured vs. predicted baPWV; D, Bland–Altman plot of measured vs. predicted ABI.
